# The Metabolic Features of Tumor-Associated Macrophages: Opportunities for Immunotherapy?

**DOI:** 10.1155/2021/5523055

**Published:** 2021-08-14

**Authors:** Sonja S. Mojsilovic, Slavko Mojsilovic, Victor H. Villar, Juan F. Santibanez

**Affiliations:** ^1^Laboratory for Immunochemistry, Institute for Medical Research, University of Belgrade, 11129 Belgrade, Serbia; ^2^Laboratory of Experimental Hematology and Stem Cells, Institute for Medical Research, University of Belgrade, 11129 Belgrade, Serbia; ^3^CRUK Beatson Institute, Glasgow G61 1BD, UK; ^4^Molecular Oncology Group, Institute for Medical Research, University of Belgrade, 11129 Belgrade, Serbia; ^5^Centro Integrativo de Biología y Química Aplicada (CIBQA), Universidad Bernardo O'Higgins, 8370993 Santiago, Chile

## Abstract

Besides transformed cells, the tumors are composed of various cell types that contribute to undesirable tumor progression. Tumor-associated macrophages (TAMs) are the most abundant innate immune cells in the tumor microenvironment (TME). Within the TME, TAMs exhibit high plasticity and undergo specific functional metabolic alterations according to the availability of tumor tissue oxygen and nutrients, thus further contributing to tumorigenesis and cancer progression. Here, we review the main functional TAM metabolic patterns influenced by TME, including glycolysis, amino acid, and fatty acid metabolism. Moreover, this review discusses antitumor immunotherapies that affect TAM functionality by inducing cell repolarizing and metabolic profiles towards an antitumoral phenotype. Also, new macrophage-based cell therapeutic technologies recently developed using chimeric antigen receptor bioengineering are exposed, which may overcome all solid tumor physical barriers impeding the current adoptive cell therapies and contribute to developing novel cancer immunotherapies.

## 1. Introduction

Tumor-associated macrophages (TAMs) and their precursors represent a large proportion of infiltrating myeloid cells in the microenvironment of most solid human malignancies, and they play a crucial role in tumorigenesis [1–3]. TAMs can be derived from blood monocytes and tissue-resident macrophages (M*Φ*s) [4] and comprise a highly dynamic and heterogeneous set of cells full of intermediate polarization phenotype. Indeed, a high degree of TAM heterogeneity is present between cancer patients and within different tumor areas of the same patient [5].

Within the tumor microenvironment (TME), O_2_ levels vary dramatically depending on blood supply and hypoxic areas are often present within a tumor tissue [6]. TAMs infiltrate hypoxic regions, in part, being attracted by several chemotactic signals secreted by cancer cells due to low oxygen pressure. Upon arrival, they suffer a reduction in motility and accumulate at ischemic tumor sites, which may explain the high TAM density in hypoxic and necrotic TME areas of some types of cancers [7, 8].

Furthermore, under different microenvironmental signals and perturbations, TAMs undergo different activation states, reflecting the capacity of these cells to acquire and move through an entire spectrum of phenotypic and metabolic functional patterns. The main M*Φ* phenotype extremes denominated as the proinflammatory M1 phenotype (or classical activation) that exhibits antitumoral functions and the anti-inflammatory M2 phenotype (or alternative activation) that possesses a protumoral phenotype [9]. Specific factors associated with inflammation, which include endotoxin, interferon- (IFN-) *γ*, and interleukin- (IL-) 1*α*, induce M1, whereas M2, which comprises M2a, M2b, and M2c subtypes, is induced by specific stimuli, including IL-4, IL-10, IL-13, transforming growth factor- (TGF-) *β*, and glucocorticoids [9–12]. Recently, an M2d subtype linked to TAMs has been proposed, induced by specific TME-associated anti-inflammatory signals, such as IL-6, Toll-like receptor (TLR) ligands, and adenosine A2 receptor (A2R) agonists. At the same time, they secrete elevated levels of IL-10, TGF-*β*, and VEGF, which contribute to tumor growth, angiogenesis, and metastasis [9]. TAMs may secrete VEGF, anti-inflammatory cytokines TGF-*β*, and IL-10, alongside immune checkpoint ligand expression, such as programmed death-ligand 1 (PD-L1). Moreover, TAMs exert T-cell immunosuppression by depleting essential amino acids via arginase-1 (ARG1) expression and recruit regulatory T-cells (Tregs) that further contribute to the inhibition of antitumor immune responses within the TME [13, 14].

In general, TAMs seem to display an M2-like polarization rather than an M1 phenotype preferentially. Nevertheless, at early tumor phases, TAMs are programmed by cancer signals into a more M1-like phenotype. Over the course of tumor progression to more advanced stages, with the establishment of inflammatory dysbalanced and immunosuppressive TME with prevalent necrotic/hypoxic areas, TAMs begin to display an anti-inflammatory M2-like phenotype [15, 16]. Furthermore, various subpopulations of TAMs have also been identified as TIE2-positive M*Φ*s [17], programmed cell death protein-1- (PD-1-) expressing TAMs [18], and C-C chemokine receptor type-2- (CCR2-) expressing TAMs [19]. In addition, it is essential to consider that, depending on nutrients, oxygen availability, and cell–matrix and cell–cell interactions in different sites of malignant tissue, TAMs can adapt their intracellular metabolism for the appropriate polarization [5, 20]. Moreover, TAM diversities require a rethinking and update of M1 and M2 nomenclature for a more accurate classification of their inflammatory phenotypes [21, 22]. Importantly, evidence supports a tumor-promoting role of TAMs and high frequencies of TAMs are generally associated with poor prognosis in most human cancers [23].

In this review, we analyzed the metabolic pathways of TAMs that allow them to adapt to oxygen and nutrient availability in the TME. This may contribute to the promotion of tumor growth and progression. Furthermore, because of the protumorigenic role of TAMs in cancer, we also present primary therapies targeting TAMs, including small drugs, combinations with immune-checkpoint inhibitors (ICI), and the current strategy of the chimeric antigen receptor (CAR) to engineer macrophages towards the adoption of antitumor functions.

## 2. Metabolic Activities in Tumor-Associated Macrophages

**Figure 1:**
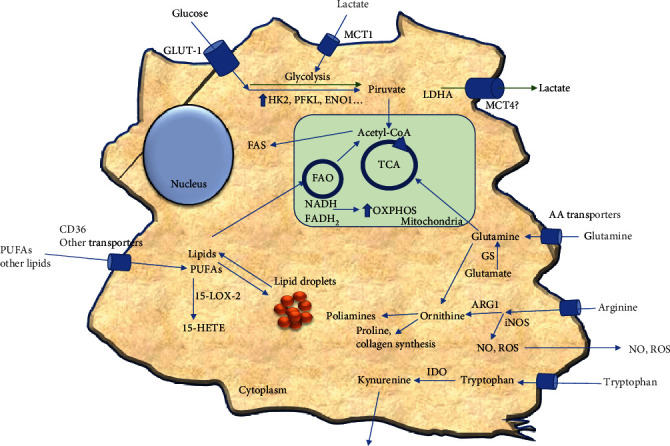
Metabolic features of tumor-associated macrophages (TAMs) may dynamically adjust their metabolism within the tumor microenvironment. Extracellular lactate can stimulate glycolysis by creating a pseudohypoxic milieu that may increase lactate production and secretion. Also, TAMs utilize glycolysis, TCA, and OXPHOS to increment the rate of bioenergetic production. TAMs exhibit increased glutamine, arginine, and tryptophan metabolism, increasing energy production, collagen synthesis, and immunosuppressive functions. Furthermore, depending on the TAM stage, lipid metabolism is also altered due to lipid uptake and storage may increase or lipids may be derived towards FAO and participate in TCA and OXPHOS. Moreover, active glycolysis may connect with F.A.S. by increasing acetyl-CoA production; for further details, see the text. TCA: tricarboxylic acid cycle; OXPHOS: oxidative phosphorylation; FAO: fatty acid oxidation; FAS: fatty acid synthesis; PUFAs: polyunsaturated fatty acids; 15-LOX-2: 15-lipoxygenase-2; 15, HETE: 15-hydroxyeicosatetraenoic acid; ARG1: arginase-1; iNOS: inducible nitric oxide synthetase; NO: nitric oxide; ROS: reactive oxygen species; IDO: indoleamine-2,3-dioxygenase; LDHA: lactate dehydrogenase A; MCT-1/4: monocarboxylate transporters-1/4; HK2: hexokinase-2; PFKL: phosphofructokinase; ENO1: enolase-1.

Solid tumors comprise complex protein and cellular components that generate a favorable ecosystem supporting transformed cell growth [24]. TAMs are recruited and infiltrate the tumor mass [25]. Moreover, TAMs contribute by promoting tumor initiation and progression, angiogenesis, and metastasis. Meanwhile, all these cancer events seem to be influenced by TAM subpopulations with relevant phenotypes corresponding to specific tumor regions, exhibiting different cytokine and metabolic profiles regulated by TME [21, 26]. TAMs dynamically adjust their metabolism to survive hostile tumor conditions and display their anti-inflammatory potential to control the tumor's dysfunctional inflammation resulting from cancer cell transformation. In general, TAM literature data indicate that TAMs possess enhanced glycolysis, fatty acid oxidation (FAO), fatty acid synthesis (FAS), and altered glutamate metabolism [25] (Figure 1).

## 3. Glucose Metabolism in TAMs

TAMs are highly dependent on aerobic glycolysis to reach TME hypoxic regions, and ATP production by glycolysis sustains the necessary cytoskeletal reorganization for cell motility [8, 27, 28]. Once TAMs arrive at hypoxic tumor regions (less than 10 mmHg, <1% oxygen), hypoxia effects impair M*Φ* migration and TAMs accumulate at ischemic tumor sites [8]. Furthermore, the expression of regulated in development and DNA damage response 1 (REDD1), an inhibitor of the mechanistic target of rapamycin (mTOR), is upregulated in hypoxic TAMs and inhibits glycolysis [29], which may affect TAM motility in hypoxic tumor regions. Consistently, dichloroacetic acid, a glycolysis inhibitor, strongly reduces macrophage migration [30]. In addition, pyruvate kinase muscle 2 (PKM2) colocalizes with F-actin in macrophage filopodia and lamellipodia during cell migration [30].

Increased aerobic glycolytic activities of TAMs have been illustrated by comparing the metabolic reprogramming of bone marrow-derived macrophages; primary TAMs were derived from the mouse mammary tumor virus promoter-driven expression of the polyomavirus middle T antigen (MMTV-PyMT) tumor model and human monocytic THP-1 cell line stimulated with tumor extract solution from breast cancer patients. Critical glycolytic enzymes hexokinase 2 (HK2), downstream phosphofructokinase (PFKL), and enolase 1 (ENO1) were increased in all situations [31]. Similarly, conditioned media from human pancreatic ductal adenocarcinoma (PDAC) cell lines induce *in vitro* TAM-like cells with upregulated *HK2*, glucose-6-phosphate isomerase (*GPI*), aldolase A (*ALDOA*), triosephosphate isomerase 1 (*TPI1*), and phosphoglycerate kinase 1 (*PGK1*) transcript expression and increased L-lactate production by expression of lactate dehydrogenase A (*LDHA*) [32]. Additionally, nonmedullary thyroid carcinoma-induced TAMs exhibit an elevated extracellular acidification rate (ECAR) and oxygen consumption rate (OCR) related to increased glucose metabolism via Akt/mTOR signaling [33]. More recently, de-Brito et al. [34] have demonstrated that TAMs show high glycolytic activity and secretion like the M1/M(LPS + IFN − *γ*) phenotype. Obtained TAMs, by treating macrophages *in vitro* with a conditioned medium of human melanoma cells, exhibit increased glucose transporter 1 (GLUT-1) expression and elevated gene expression of HK2 alongside increased AKT/mTOR signaling. In line with Arts et al. [33], these TAMs also present high basal and maximal OCR and high mitochondrial ATP production, thus performing oxidative phosphorylation (OXPHOS) similarly to M2/M(IL-4). Interestingly, the TAM maintenance phenotype depends on glycolysis and is independent of both OXPHOS and pentose phosphate pathways (PPP) [34].

Cancer cells metabolize glucose mainly via aerobic glycolysis, known as the “Warburg effect,” leading to high lactate concentrations in the TME [35]. For instance, thyroid cancer-derived lactate enhances the aerobic glycolysis of endogenous TAMs mediated by the AKT1/mTOR pathway and stabilizes their protumor phenotype [32]. CD163^+^ TAMs correlate with LDHA2 expression in bladder cancer, head and neck cancer, and malignant skin cancer [8]. Meanwhile, M*Φ* uptake of cancer cell-derived lactate can be performed via monocarboxylate transporter-1 (MCT1) on the cell surface and be facilitated by a local low pH [36]. Afterwards, intracellular lactate can be metabolized to pyruvate again by LDH1, which competes with *α*-ketoglutarate to negatively regulate prolyl hydroxylase, thus preventing hypoxia-inducible factor- (HIF-) 1*α* ubiquitination and proteasomal degradation and promoting a glycolytic pathway [37]. An elevated glucose consumption rate by TAMs is also potentially immunosuppressive since glucose-depleted TME reduces antitumor T-cell activities [14]. In addition, TAMs present a low PPP pathway activity and it seems to be nonessential for M2-like phenotype marker expression and TAM functions [34]. Furthermore, TAMs also present increased OCR and OXPHOS activities to produce high amounts of ATP [33, 34], which is in line with the M2 macrophage phenotype being related to an intact tricarboxylic acid cycle (TCA) and increased OXPHOS activity [9].

## 4. Amino Acid Metabolism

Nutrients and metabolic waste products may alter the TAM phenotype and functions [38]. For instance, tumor-derived lactate potently induces ARG1 in TAMs via ERK1, 2/STAT3, and HIF-1*α* stabilization. Additionally, this “*pseudo-hypoxic*” HIF-1*α* activation by tumor metabolites enhances proangiogenic TAM function that fosters tumor growth [39–41]. ARG1^+^ TAMs may promote tumor growth as demonstrated by the fact that M*Φ*-overexpressing ARG1 enhances tumor cell proliferation, while ARG1-deficient M*Φ*s are associated with reduced tumor size in mice models [39, 42]. A potential mechanism has been proposed for ARG1 tumor-promoting functions. ARG-1 may promote cancer cells by enhancing polyamine production, stimulating M2 gene expression, and stabilizing TAMs' protumorigenic phenotype [43, 44]. Although TAMs seem to have a low NO production capacity, as is observed in murine mammary and human ovarian tumors, consistent with M2-like and protumor characteristics [44–46], simultaneous ARG1 and inducible nitric oxide synthase (iNOS) pathways have been observed in tumor-licensed TAMs [47]. ARG1 and iNOS coexpression at a low arginine concentration may favor reactive oxygen species (ROS) and reactive nitrogen species (RNS) production, thus further inhibiting intratumoral T-cell functions. This dissimilarity reinforces phenotype differences between TAMs and M2-polarized macrophages since, in ischemic tumor domains, TAMs may coexpress ARG1 and iNOS/NOS2, representing an intermediary M1/M2 stage [9].

TAM dependence on the glutamine-glutamate pathway is an essential metabolic characteristic. TAMs isolated from glioblastomas and glioblastoma cell-induced TAMs exhibit increased glutamate transport genes and cellular metabolism expression. qPCR analysis indicated an increased expression of glutamate receptor 2 (GRIA2), glutamate transporters GLT-1 (SLC1A2) and GLAST (SLC1A3), and glutamine synthetase (GS) and a decreased expression of cysteine glutamate antiporter, which may improve TAM resistance to glutamine starvation. Glutamate and glutamine can be used for energetic requirements in TAMs, while glutamine can also be released to the tumor extracellular milieu to fuel cancer cells [44, 48]. Additionally, thyroid cancer-induced TAMs show a reduced glutamate concentration due to a potential utilization by glutamine metabolism to replenish the TCA cycle [43]. In addition, targeting glutamine metabolism by JHU083, a prodrug that broadly inhibits glutamine-metabolizing enzymes, reprograms TAMs towards the M1-like phenotype and enhances antitumor immunity without affecting their total number within the tumor [49]. Moreover, targeting GS in M2 and TAMs can also shift macrophage polarization towards an M1-like phenotype. GS inhibition leads to HIF-1*α* activation, promotes succinate accumulation and glucose-dependent metabolism, subverts T-cell immunosuppression, and prevents metastasis [50].

In addition, TAMs may strongly express IDO that contributes to creating an immunosuppressive microenvironment by tryptophan deprivation. In fact, the conditionate medium of IDO^+^ TAMs suppressed T-cell response, while pretreatment of TAMs with an IDO inhibitor restored T-cell proliferation [51]. Besides the local tryptophan reduction, IDO also catabolizes tryptophan to kynurenine, which negatively influences T-cell proliferation and immune responses [52].

## 5. Fatty Acid Metabolism in TAMs

Tumor tissues may have aberrant activation of *de novo* lipogenesis due to an overexpression of fatty acid synthase (FASN), ATP citrate lyase (ACLY), and acetyl-CoA carboxylase (ACC), which is associated with unfavorable cancer outcomes. FAS inhibition suppresses transformed cell growth, while increased lipid synthesis promotes cancer cell proliferation by a continuous substrate supply for cellular membrane generation and bioenergy production [44, 53, 54].

Lipid metabolism may play a role in shaping the functional phenotype in TAMs in the TME. FAS is vastly increased at the transcriptome level in thyroid carcinoma- (TC-) induced TAMs. Moreover, TC-induced TAMs display increased glucose metabolism and increased intracellular levels of acetyl-CoA, which can be used for fatty acid synthesis. Furthermore, mTOR pathway activity and HIF-1*α* expression are elevated in TAMs [33]. In addition, mTORC1 signaling is implicated in lipid, protein, and nucleotide syntheses. Thus, mTORC1 mediates metabolic reprogramming and differentiation in macrophages [38, 55].

Interestingly, TAMs isolated from ovarian carcinoma patients show a PPAR*β*/*δ*-dependent elevated transcriptome due to high polyunsaturated fatty acid (PUFA) levels in the TME. Specifically, linoleic acid and arachidonic acid are stored in highly stable lipid droplets in TAMs and may contribute to TAM polarization [56, 57]. In addition, TAMs derived from renal carcinoma produce elevated levels of eicosanoids via the activation of 15-lipoxygenase- (LOX-) 2 (15-LOX-2) and release substantial amounts of the arachidonic acid metabolite 15-hydroxyeicosatetraenoic acid (15-HETE) [58]. LOXs are nonheme iron-containing dioxygenases that catalyze the stereospecific peroxidation of PUFAs to the corresponding hydroperoxyl derivatives [59]. Beyond its role in regulating lipid homeostasis, 15-LOX-2 also contributes to an increased TAM expression of CCL2 chemokine and IL-10, leading to a local immune tolerance within the TME [58, 60].

Moreover, several studies have shown that cellular accumulation of lipids is crucial in regulating the function of TAMs. Macrophages accumulate lipids within the TME and support immunosuppression. Both *in vitro* cancer-stimulated macrophages and TAMs isolated from different tumor models exhibit an increased expression of ABHD5, a lipolytic factor of triglycerides, whereas monoacylglycerol lipase (MGLL) is downregulated. Meanwhile, M*Φ*s, derived from a transgenic mouse model with a specific overexpression of MGLL (Tg^MGLL^) in myeloid cells, were refractory to accumulated lipids in response to cancer cell stimuli. Furthermore, MGLL downregulation contributes to the suppression of tumor-associated CD8^+^ T-cell function and tumor progression. In addition, myeloid MGLL overexpression potentiates M1-like TAM expansion to the detriment of M2-like TAMs. Thereby, the TAM phenotype requires a reduced MGLL expression to stabilize and display immunosuppressive and tumor promoter functions [61].

TAMs from both human and murine tumor tissues accumulate lipids that rely on elevated levels of CD36 expression and ensure substrates to FAO and OXPHOS for energy production. Lipid accumulation in TAMs correlates with the upregulation of genes involved in fatty acid *β*-oxidation, such as CPT1A, an FAO rate-limiting enzyme, acyl-CoA dehydrogenase medium-chain (ACADM), hydroxyacyl-CoA dehydrogenase/3-ketoacyl-CoA thiolase/enoyl-CoA hydratase (a trifunctional protein), and hydroxyacyl-CoA dehydrogenase (HADH), without any modifications of gene expression involved in glucose metabolism. Conversely, reduced lipid uptake in CD36-KO M*Φ*s or FAO inhibition by etomoxir can prevent the generation of TAMs and reduce their protumor activities [62].

Interestingly, cell death-associated factors may regulate TAM generation and fatty acid metabolism. For example, TAM-like cells, generated from THP-1 M*Φ*s cocultured with MCF-7 tumor cells, depend on caspase-1-dependent peroxisome proliferator-activated receptor *γ* (PPAR *γ*) cleavage. When truncated PPAR*γ* translocates to mitochondria to interact and inhibit medium-chain acyl-CoA dehydrogenase (MCAD), it results in FAO inhibition and consequently induces lipid droplet accumulation and TAM differentiation [63]. On the other hand, receptor-interacting protein kinase 3 (RIPK3), a central factor in necroptosis, is downregulated in hepatocellular carcinoma- (HCC-) associated TAMs. RIPK3 reduction provokes an inhibition of caspase1-mediated cleavage of PPAR*γ* that promotes FAO pathway activation and M2 polarization within the TME. Consistently, RIPK3 overexpression or FAO blockade prevents TAM-induced immunosuppression and impairs HCC tumorigenesis [64]. These data suggest a time-dependent relation of caspase-1 activation and RIPK3 downregulation to balance lipid storage and degradation according to TAM's energy needs in tumor tissues. Intriguingly, human TAMs, induced with a conditioned medium of human melanoma, demonstrate a low absorption of exogenous palmitate/BODIPY FLC16, reduced CD36 expression, and FAO activity similar to M1 M*Φ*.

Nevertheless, these determinations were done in environments with an atmospheric oxygen concentration, where glucose in culture media may be the primary fuel source and the TAM phenotype maintenance depends on the glycolytic pathway. Moreover, no determination of CD36 or lipid uptake was performed under hypoxic conditions [34]. In summary, lipid uptake, intracellular lipid accumulation, FAS, and FAO demonstrate TAMs' metabolic flexibility and adjustment to achieve a proinflammatory and protumorigenic program to sustain tumor development and malignancy.

## 6. TAMs and Immunotherapy Perspectives

The innate immune system cells that interplay with the adaptive immune system are essential in preventing the progression and acquisition of malignancy stages of transformed cells [65, 66], and a crucial subject in antitumor immunology is the struggle against of the immunosuppressive environment within the tumor stroma producing dysfunctional antitumor responses. TAMs exert protumoral functions that enhance tumor progression, tumor growth, and neoangiogenesis and facilitate the establishment of the immune-suppressive microenvironment, making them attractive therapeutic targets for cancer therapy [26]. Therefore, targeting TAMs may provide novel treatment options to cancer types currently unresponsive to conventional chemotherapeutics and synergize with current immunotherapies [67].

### 6.1. TAMs and Immune Checkpoint Inhibitors

**Figure 2:**
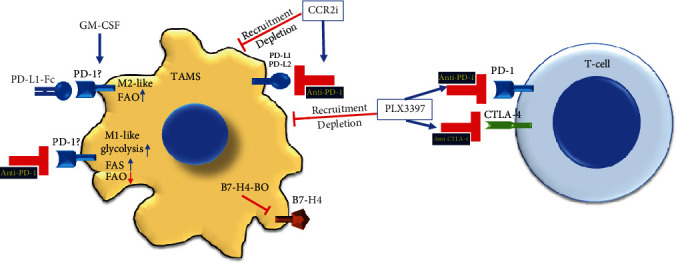
Examples of TAM combinatory targeting with immune checkpoint inhibitors. TAM depletion and recruitment inhibition to the tumor microenvironment, by CCR2 inhibitor or PLX3397, potentiate immune checkpoint inhibition by anti-PD-1, anti-PD-L1/2, or anti-CTLA-4 and further stimulate T-cell functions. Similarly, inhibition of B7-H4 by specific morpholino antisense oligonucleotides (B7-H4-BO) contributes to T-cell stimulation, tumor regression, and tumor growth inhibition. In addition, under GM-CSF-induced macrophages, increased PD-1 expression, and manipulation either with PD-1 agonist (PD-L1-Fc) or with anti-PD-1 may favor the M2-like phenotype or M1-like phenotype, respectively, alongside metabolic reprogramming, which needed to be confirmed in TAMs. PD-1: programmed death protein-1; PD-L1: programmed death protein-ligand-1; CTLA-4: cytotoxic T-lymphocyte antigen 4; FAO: fatty acid oxidation; FAS: fatty acid synthesis; GM-CSF: granulocyte-macrophage colony-stimulating factor.

One of the main TAM mechanisms involved in cancer support and promotion is their potent capacity to create an immune-tolerant TME by preventing immune cytotoxic cell effector functions. TAMs may suppress antitumor immune responses; they can activate adverse regulatory pathways or checkpoints associated with immune homeostasis and can allow cancer cells to actively escape from immunosurveillance [26, 68]. Recent studies have indicated that TAMs express B7 family ligands PD-L1 (CD274) and PD-L2 as well as CD80 (B7-1) and CD86 (B7-2), which bind to inhibitory receptors PD-1 (CD279) and cytotoxic T-lymphocyte antigen 4 (CTLA-4 or CD152) and induce CD8^+^ T-cell dysfunction [66]. PD-1 and CTLA-4 are expressed in activated immune effector cells as part of regulatory and safety mechanisms to control the resolution phase of immune response and inflammation [26]. Furthermore, TAMs also express B7 family checkpoint ligands with direct suppressive effects on tumor-infiltrating T-cells, such as the coinhibitory molecule B7-H4 and V domain immunoglobulin suppressor of T-cell activation (VISTA, B7-H5), which bind CD28H [69, 70]. B7-H4 binding to an unknown target-cell receptor inhibits T-cell proliferation, cell cycle progression, and cytokine production [71, 72]. In contrast, B7-H4 depletion, by using a B7-H4-specific morpholino antisense oligonucleotides (B7-H4 blocking oligos), switches TAMs to T-cell-stimulating functions alongside with tumor regression and tumor growth (Figure 2) [69]. TAMs may directly inhibit T-cell functions via these immune checkpoint ligands and reduce immune checkpoint therapy efficacy [73].

Although ICIs have shown great potential, due to their therapeutic success in several cancers, such as melanoma and lung cancers, and leukemias, the percentage of effectiveness still does not fulfill the expected outcomes in cancer immunotherapy [66, 74]. TAMs, the highest in abundance among tumor-infiltrating immune cells, exert multifaceted roles in promoting tumor progression, and it has been suggested that the selective targeting of TAM functions in combination with ICI therapies may synergize with current cancer immunotherapies [75].

For instance, targeting colony-stimulating factor (CSF) 1, a TAM recruitment factor, improves the therapeutic effect of checkpoint inhibitors. CSF1R inhibition by PLX3397 reduces TAMs' tumor infiltration and their immunosuppressive phenotypes, potentiates ICI effects against PD-1 and CTLA4, and impairs tumor expansion by approximately 50%. Moreover, this combinatory approach induces about a 15% regression in established pancreatic cancer tumors [76]. In line with these results, CCR2 antagonists increment anti-PD-1 antibody efficacy and reduce TAM accumulation within tumor tissues [77] (Figure 2). In addition, colon carcinoma CT26 murine syngeneic tumors that exhibit high immunogenicity and the mesenchymal-like phenotype are susceptible to immunotherapy with anti-PD-L1 mIgG2, an IgG subclass with the highest affinity for activating Fc*γ*R typically expressed by TAMs. Anti-PD-L1 mIgG2 antitumor therapy results in a significant inhibition of tumor growth and improved long-term mouse survival. This tumor inhibition was due to the anti-PD-L1 mIgG2 potential to directly target tumor-associated myeloid cells expressing Fc*γ*R [78].

Similarly, the antibody targeting macrophage receptor with the collagenous structure (MARCO), by crosslinking the inhibitory FcgRIIB on TAMs, synergistically increases anti-CTLA4 immunotherapy in melanoma and colon cancer mouse models. Interestingly, anti-MARCO treatment results in repolarization of the M2-like anti-inflammatory TAM population to M1-like proinflammatory TAMs that increase tumor immunogenicity [79]. Thus, it is believed that preventing or rescuing TAMs from immunosuppressive functions, either by depletion or phenotype repolarization, may significantly improve ICI immunotherapies by reversing immune dysfunction and restoring the cytotoxic antitumor function of T lymphocytes within the TME. Several ongoing clinical trials have considered combinatorial TAMs and immune checkpoints targeting and are reviewed elsewhere [26, 80].

Notably, in chronic lymphocytic leukemia- (CLL-) derived monocytes, triggering the PD-1 checkpoint by using the bioactive recombinant PD-L1 protein hampers glycolysis (reduces glucose uptake, glucose transporters, and expression of glycolytic molecules) and shifts their metabolism toward OXPHOs, which may suggest the PD-1/PD-L1 axis as a novel immune metabolic player in myeloid cells [81, 82].

Although no data exist about ICI on TAM metabolism, some information can be taken from other models. Trophoblast–M*Φ* interaction during early pregnancy may illustrate molecular pathways in the PD-1/PD-L1 axis-mediated regulation of myeloid cells. The treatment of normal monocytes with granulocyte-macrophage- (GM-) CSF significantly increases PD-1 mRNA expression. Afterwards, PD-L1 agonist (PD-L1 Fc) engages M*Φ*s expressing PD-1 and triggers the polarization of GM-CSF-differentiated M*Φ*s towards the M2 phenotype with increased FAO activity. The anti-PD-1 blocking antibody promotes macrophage polarization towards the M1 phenotype with enhanced glycolytic metabolism, reduced FAO, and increased fatty acid synthesis pathway. These events occur alongside increased PI3K/AKT/mTOR and MEK/ERK activity pathways. Thus, PD-1 signaling might modulate macrophage polarization via reprogramming their metabolism [83]. PD-1 expression has also been observed in other M*Φ* models, playing a suppressive role associated with M2 polarization and increasing with the stage of disease in colorectal cancer patients [18, 84].

Nevertheless, further investigations addressing the immune checkpoint regulation of TAM metabolism are required, as they would contribute to an understanding of the complex immune-metabolic network in the TME.

### 6.2. TAMs and Adoptive Cell Therapy

**Figure 3:**
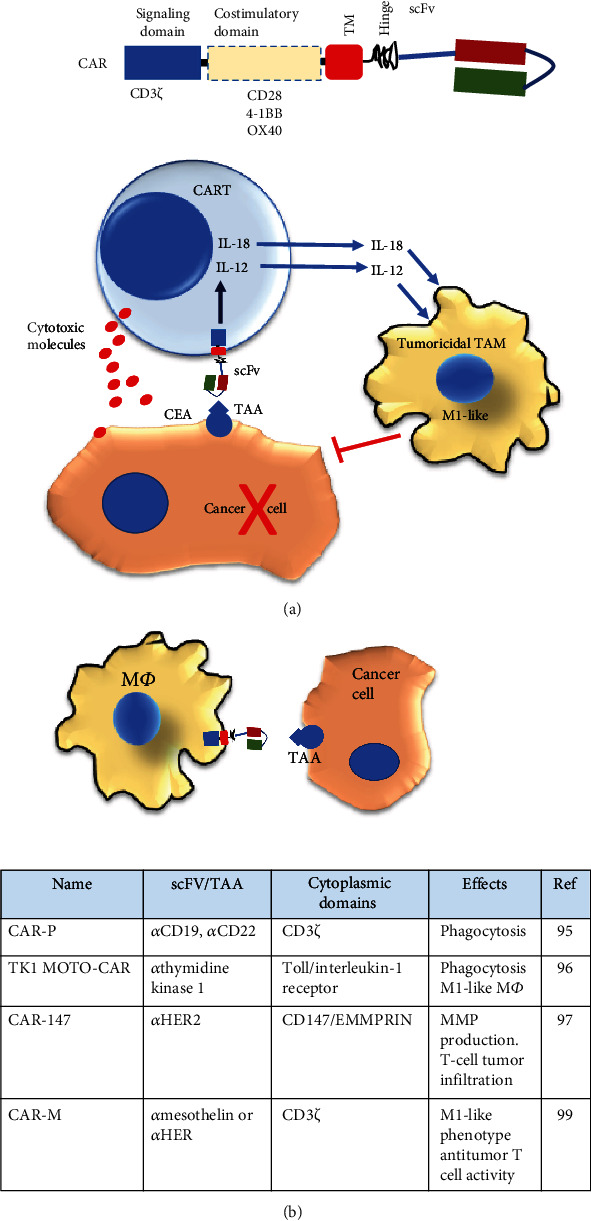
Adoptive cell therapy and macrophage. (a) Primary chimeric antigen receptor (CAR) features are exposed. scFv: single-chain antibody variable fragment; TM: transmembrane domain. The bottom figure indicates the treatment with the fourth-generation CAR T-cell strategy. After engagement to tumor-associated antigen (TAA), CAR T-cells are activated and may exert antitumor activities by releasing cytotoxic molecules. Furthermore, CAR activation promotes the expression and secretion of IL-12 or IL-18 that may induce TAM shift to tumoricidal functions. (b) TAMs engineered with CAR new strategies implicate the CAR-modified macrophages. The table indicates CAR-modified macrophages, specific targets, intracellular CAR domains, and the main functional activities promoted by CAR macrophage engineering.

A new therapeutic technique has recently emerged, CAR T-cells, which provides new insight into adoptive cell therapy (ACT) for cancer. The CAR construct results from fusing a specific anti-tumor-associated antigen (TAA) antibody polypeptide chain with the TCR/CD3*ζ* signal-mediated activating machinery of the T-cell. Additionally, it may contain one or more domains derived from costimulatory T-cell receptors CD28, 4-1BB, or OX40 (Figure 3(a)). The TAA-CAR-specific construct is ectopically expressed in the immune effector cells to recognize and potentially target cancer cells. When CAR T-cells bind to TAA at the cancer cell surface, they proliferate and kill tumor cells. Thus, CAR T-cells represent a significant advancement in cancer immunotherapy and a genetic engineering platform to develop CAR-based immunotherapies using other immune cells [85, 86]. Remarkably, fourth-generation CAR-T-cells (TRUCKs), engineered with anti-carcinoembryonic antigen (CEA, CD66) antibody single-chain peptide and modified to secrete inducible IL-12 (IL-12), upon engaging tumor cells, reprogram TAMs recruited into the tumors to be tumoricidal cells that cooperate with CAR-T-cells for tumor regression and cancer cell elimination (Figure 3(a)) [87, 88].

Similarly, CAR T-cells releasing inducible IL-18, upon CAR stimulation, change the TME immune cell landscape into an acute inflammatory feature, owing to an increased frequency of M1-like TAMs and the improved infiltration of activated dendritic cells and natural killer cells. As a result, an improvement of mice survival with advanced pancreatic and lung tumors has been observed, as has antigen-specific memory [89].

Although CAR T-cells have significant success in treating hematological malignancies, targeting solid tumors is challenging to implement for ACT due to impediments to trafficking and infiltrating tumor tissues [90]. Beyond functioning as professional antigen-presenting cells (APC) and actively participating in the immune response, M*Φ*s possess a high capacity to migrate and infiltrate tumors in response to tumor-secreted chemokines [91, 92]. This tumor-infiltrating capacity makes M*Φ*s strong candidates as vehicles for CAR therapy (Figure 3(b)). In this sense, the concept of CAR modification in M*Φ*s has recently been introduced. Several CARs for CD19- or CD22-targeted phagocytosis were engineered for M*Φ* cell transfection and named CAR-Ps. The CAR-P molecules contain the extracellular single-chain antibody variable fragment (scFv) recognizing the B cell antigen CD19 or CD22 and the CD8 transmembrane domain as in the ɑCD19/*α*CD22 CAR-T, as well as cytoplasmic domains Megf10, the common *ɣ* subunit of Fc receptors (FcR*ɣ*), or CD3*ζ*, and could promote phagocytosis. These CAR-Ps possess excellent specificity and a phagocytotic ability for both CD19- and CD22-coated beads and for Raji B cells *in vitro*. This study demonstrates that, beyond T-cell activation, the CAR strategy is transferrable to phagocytic cells and sufficient to promote specific engulfment and eliminate cancer cells. Tumor antigen-specific CARs make M*Φ*s applicable in ACT to target solid tumors [93].

Another CAR-modified M*Φ* strategy considers a Toll-like receptor-chimeric antigen receptor expression. In this case, the chimeric protein is the result of the fusion of the Toll/interleukin-1 receptor (TIR) signaling domain for intracellular signal transduction and scFv for thymidine kinase 1 targeting (TK1 MOTO-CAR cells). The in vitro analysis indicates that TK1 MOTO-CAR cells exhibit an M1-skewed phenotype and phagocytic activity. Moreover, these CAR cells specifically induce cell death and form clusters around the TK1-positive non-small cell lung carcinoma NCI-H460 cell line [94, 95].

Furthermore, M*Φ*s are modified with anti-hHER2 scFv and the transmembrane and intramembrane regions of the mouse CD147 molecule construct (CAR-147). CD147 (also named EMMPRIN) is a well-known extracellular matrix metalloproteinase (MMPs) inducer [96]. When cocultured with HER2-4T1 cells, the expression of MMP3, MMP9, MMP10, MMP11, MMP12, MMP13, and MMP14 in CAR-147 M*Φ* is significantly upregulated. Although CAR-147 M*Φ*s did not inhibit cancer cell growth *in vitro*, they significantly inhibited tumor growth in the 4T1 breast cancer mouse model. Moreover, these M*Φ*s promoted T-cell infiltration into the tumor concomitantly with the degradation of the dense collagen-based matrix that surrounds it. Furthermore, CAR-147-expressing human M*Φ* facilitated T-cell infiltration in a three-dimensional multicellular sphere model of human breast cancer [97].

Recently, the term CAR-M has been coined regarding human M*Φ* transduced with CARs encoding the CD3*ζ* intracellular domain to target the tumor antigen mesothelin or HER2. CAR-M cells display tumor antigen-specific phagocytic activity while exhibiting a proinflammatory M1-like phenotype and promote antitumor T-cell activity [98]. Moreover, the investigational new drug application for anti-human HER2-CARM (CT-0508) was approved last year by the FDA to target recurrent or metastatic HER2-overexpressing solid tumors [99]. M*Φ*s engineered with CAR molecules seem to preferentially exhibit a proinflammatory M1-like TAM phenotype. According to the metabolic adjustment of macrophages to TME signaling, oxygen, nutrient levels, and intratumor localization, it is plausible that the CAR modification of M*Φ*s may influence glucose and nutrient flow to a more glycolytic and FAS metabolic M1-like profile.

## 7. Conclusions

Tumor metabolic activities are critical for tumor growth and progression. Cancer cells may create a dysfunctional microenvironment, producing a network of inflammatory factors, oxygen, nutrients, and metabolites that may alter the immune system response and make it protumorigenic. TAMs are one of the critical noncancer cell populations that infiltrate tumors. They actively interact with cancer cells and may promote tumor growth by regulating antitumor immune function. Moreover, TAMs dynamically adjust their metabolism according to different signals and interactions, depending on intratumor localization. Moreover, TAM-produced metabolites exert multiple biological effects in the whole TME. Thus, the success of tumor development and growth may depend on active TAM–TME metabolic crosstalk.

Due to the importance of TAMs in tumor progression, several therapeutic strategies have been investigated, from elimination to utilization as antitumor drug cargoes, including their shifting to proinflammatory and antitumoral phenotypes. All these therapies may also be combined with the current ICI immunotherapies. Furthermore, TAMs seem to be excellent candidates for adoptive cell therapy, especially as part of CAR technology. Moreover, an evaluation of metabolic flux in TAMs and metabolism interventions might further improve tumor immunotherapy. Nonetheless, more investigations are needed to elucidate TAMs' metabolic adaptation to current immunotherapies. Therefore, the metabolic manipulation of TAMs may allow for the generation of more accurate oncotherapies and increment the life quality of cancer patients.

## Data Availability

The material supporting the conclusion of this review has been included within the article.

## Abbreviations

ACC: Acetyl-CoA carboxylase
ACADM: Acyl-CoA dehydrogenase medium chain
A2R: Adenosine A2 receptor
ACT: Adoptive cell therapy
ALDOA: Aldolase A
APC: Antigen-presenting cells
ARG1: Arginase-1
ACLY: ATP citrate lyase
CEA: Carcinoembryonic antigen
CAR: Chimeric antigen receptor
CCR2: C-C chemokine receptor type-2
CLL: Chronic lymphocytic leukemia
CSF: Colony-stimulating factor
CTLA-4: Cytotoxic T-lymphocyte antigen 4
ENO1: Enolase 1
ECAR: Extracellular acidification rate
FcR: Fc receptors
FAO: Fatty acid oxidation
FAS: Fatty acid synthesis
FASN: Fatty acid synthase
GM: Granulocyte-macrophage
GPI: Glucose-6-phosphate isomerase
GLUT-1: Glucose transporter 1
GRIA2: Glutamate receptor 2
SLC1A2: Glutamate transporters GLT-1
GS: Glutamine synthetase
HK2: Hexokinase 2
15-HETE: 15-Hydroxyeicosatetraenoic acid
HADH: Hydroxyacyl-CoA dehydrogenase
HCC: Hepatocellular carcinoma
HIF: Hypoxia-inducible factor
iNOS: Inducible nitric oxide synthase
IFN: Interferon
IL: Interleukin
ICI: Immune-checkpoint inhibitors
LDHA: Lactate dehydrogenase A
15-LOX-2: 15-Lipoxygenase-2
M*Φ*s: Macrophages
MMP: Matrix metalloproteinase
mTOR: Mechanistic target of rapamycin
MCAD: Medium-chain acyl-CoA dehydrogenase
MCT1: Monocarboxylate transporter-1
MGLL: Monoacylglycerol lipase
MMTV-PyMT: Mouse mammary tumor virus promoter-driven expression of polyomavirus middle T antigen
OXPHOS: Oxidative phosphorylation
OCR: Oxygen consumption rate
PDAC: Pancreatic ductal adenocarcinoma
PPP: Pentose phosphate pathways
PPAR: Peroxisome proliferator-activated receptor
PFKL: Phosphofructokinase
PGK1: Phosphoglycerate kinase 1
PUFAs: Polyunsaturated fatty acids
PD-1: Programmed cell death protein-1
PD-L1: Programmed cell death-ligand 1
PKM2: Pyruvate kinase muscle 2
RNS: Reactive nitrogen species
ROS: Reactive oxygen species
RIPK3: Receptor-interacting protein kinase 3
REDD1: Regulated in development and DNA damage response 1
scFv: Single-chain antibody variable fragment
TGF-*β*: Transforming growth factor-*β*
TCA: Tricarboxylic acid cycle
TC: Thyroid carcinoma
TPI1: Triosephosphate isomerase
TLR: Toll-like receptor
TAA: Tumor-associated antigen
Tregs: Regulatory T-cells
TRUCKs: Fourth-generation CAR-T-cells
TAMs: Tumor-associated macrophages
TME: Tumor microenvironment
VISTA: V domain immunoglobulin suppressor of T-cell activation.
